# Detection of Acetaminophen in Groundwater by Laccase-Based Amperometric Biosensors Using MoS_2_ Modified Carbon Paper Electrodes

**DOI:** 10.3390/s23104633

**Published:** 2023-05-10

**Authors:** Marcela Herrera-Domínguez, Koun Lim, Iris Aguilar-Hernández, Alejandra García-García, Shelley D. Minteer, Nancy Ornelas-Soto, Raúl Garcia-Morales

**Affiliations:** 1 Laboratorio de Nanotecnología Ambiental, Escuela de Ingeniería y Ciencias, Tecnológico de Monterrey, Ave. Eugenio Garza Sada 2501, Monterrey 64849, NL, Mexico; 2 Department of Chemistry and Materials Science & Engineering, University of Utah, Salt Lake City, UT 84112, USA; 3 Laboratorio de Síntesis y Modificación de Nanoestructuras y Materiales Bidimensionales, Centro de Investigación en Materiales Avanzados S.C., Unidad Monterrey, Parque PIIT, Apodaca 66628, NL, Mexico; 4 Centro de Nanociencias y Nanotecnología, Universidad Nacional Autónoma de México, Carretera Tijuana-Ensenada Km. 107, Ensenada 22860, BC, Mexico

**Keywords:** electrochemical biosensor, acetaminophen, laccases, MoS_2_, emerging pollutants

## Abstract

The use of enzyme-based biosensors for the detection and quantification of analytes of interest such as contaminants of emerging concern, including over-the-counter medication, provides an attractive alternative compared to more established techniques. However, their direct application to real environmental matrices is still under investigation due to the various drawbacks in their implementation. Here, we report the development of bioelectrodes using laccase enzymes immobilized onto carbon paper electrodes modified with nanostructured molybdenum disulfide (MoS_2_). The laccase enzymes were two isoforms (LacI and LacII) produced and purified from the fungus *Pycnoporus sanguineus* CS43 that is native to Mexico. A commercial purified enzyme from the fungus *Trametes versicolor* (TvL) was also evaluated to compare their performance. The developed bioelectrodes were used in the biosensing of acetaminophen, a drug widely used to relieve fever and pain, and of which there is recent concern about its effect on the environment after its final disposal. The use of MoS_2_ as a transducer modifier was evaluated, and it was found that the best detection was achieved using a concentration of 1 mg/mL. Moreover, it was found that the laccase with the best biosensing efficiency was LacII, which achieved an LOD of 0.2 µM and a sensitivity of 0.108 µA/µM cm^2^ in the buffer matrix. Moreover, the performance of the bioelectrodes in a composite groundwater sample from Northeast Mexico was analyzed, achieving an LOD of 0.5 µM and a sensitivity of 0.015 µA/µM cm^2^. The LOD values found are among the lowest reported for biosensors based on the use of oxidoreductase enzymes, while the sensitivity is the highest currently reported.

## 1. Introduction

Water pollution is a global environmental concern, and recently particular attention has been paid to a group of organic pollutants found in low concentrations (ng/L to mg/L) [1], as they may represent a real hazard to aquatic ecosystems due to their bioaccumulation and long-term effects. These pollutants are known as contaminants of emerging concern (CEC), and in many cases they are not regulated by environmental laws. Therefore, it is of vital importance to investigate the possible effects of these pollutants at the concentrations found in the environment [2].

The term CEC includes pharmaceutical active compounds (PhACs). Among these compounds, pharmaceuticals sold without a medical prescription are especially concerning since they are more frequently discharged into water bodies either directly or indirectly [3]. Acetaminophen (ACE), also known as paracetamol, is a widely used analgesic whose presence has been detected in aquatic ecosystems [4]. ACE is the first step in pain management according to the World Health Organization (WHO) ladder [5] and has been commonly recommended for many clinical practice guidelines since its inception in the 1950s [6]. It has been estimated that the annual production of ACE is as high as 145,000 t [7], and indicators have ranked its usage in countries such as the United States (5790 t in 2002) and France (3303 t in 2005) in the top 10 [8].

Several studies have demonstrated that ACE may interfere with the endocrine system of fish, causing abnormal embryonic development, growth, and have negative effects in reproduction; furthermore, possible deleterious effects in kidney and liver have been found [9,10]. ACE has been found worldwide in the environment in concentrations up to 230 µg/L [8,11]. Due to these reasons, some organizations, such as the Minnesota Department of Health (MDH) in the US, have determined a guidance value for acetaminophen in drinking water of 200 µg/L, since the liver is the most sensitive organ to ACE exposure [12].

ACE has been quantified in environmental samples using well-established techniques such as chromatography, spectroscopy, and capillary electrophoresis. Nevertheless, these present some drawbacks, such as high costs, long analysis times, and exhaustive sample preparation steps [13,14,15]. Therefore, the development of environmental electrochemical biosensors has been increasing recently due to their advantages, such as short response times, minimal sample preparation, and relatively easy construction and operation coupled with high sensitivity and selectivity [16]. Among electrochemical biosensors, enzymatic-based biosensors have shown advantages in the detection of a wide variety of chemical compounds. These devices consist of a transducer and a biological component. In enzyme-based biosensors, the enzyme chemically interacts with the target compound, and this interaction is then transduced into a measurable signal [17,18,19]. Moreover, it is important to note that the determination of ACE by electrochemical sensors has been mostly studied in pharmaceuticals and human body fluids, and few studies have been conducted on environmental samples [9].

In fact, since these types of electrochemical sensors still have some limitations when being used in real environments, it has become necessary to use other technologies and approaches, such as the development of conductive polymers, the use of nanomaterials, and the obtaining of new biological components, to improve their electrochemical response. Among the biological elements being developed, laccase enzymes are multicopper oxidoreductases that catalyze the oxidation of phenolic and amino aromatic-like compounds into their oxidized form, accompanied by the reduction of molecular oxygen [20,21,22]. Furthermore, the use of laccases provides other advantages compared to other oxidoreductases, such as biocatalysis without additional cofactors, good thermostability, stability to pH, and denaturing substances [23]. Specifically, two laccase isoforms from the native strain *Pycnoporus sanguineus CS43*, LacI and LacII, have been demonstrated to possess high resistance to inhibitors and thermal stability up to 60 °C [24]. According to previous studies, both isoforms are promising recognition elements for electrochemical biosensors, since they have exhibited an onset potential of over +650 mV vs. Ag/AgCl at pH 4 [25].

On the other hand, nanostructured electrochemical transducers can be used to improve a biosensor [18,26], as these materials can exhibit enhanced conductivity and catalytic activity [27,28,29]. Molybdenum sulfide (MoS_2_) presents interesting structural, physicochemical, thermal, mechanical, and electrocatalytic properties (10–15 mA/cm^2^) [30,31], and has been employed in the development of a series of sensors for the detection of analytes such as NO in as gas sensor, glucose, DNA, dopamine, and bisphenol A in biological and pharmaceutical samples [27,32,33,34,35,36].

In this work, a laccase-based biosensor for the environmental determination of acetaminophen in water was developed. The biosensor comprises carbon paper functionalized with MoS_2_, a two-dimensional nanomaterial, and laccase enzyme. The performance of purified laccase isoforms from the native *Pycnoporus sanguineus* CS43 fungus was compared to a commercially available laccase from *Trametes versicolor* (TvL). This is the only work in which a laccase-based biosensor has been specifically developed for the environmental determination of ACE in water. ACE quantification was evaluated in real samples (i.e., a pool of 31 groundwater samples from northern Mexico), and the pharmaceutical was successfully detected at environmentally relevant concentrations. Moreover, high sensitivity was achieved due to the presence of MoS_2_.

## 2. Materials and Methods

### 2.1. Reagents

Acetaminophen (ACE), potassium hydroxide, dibasic sodium phosphate, ammonium heptamolybdate tetrahydrate (AHMo), thiourea (TU), molybdenum (VI) oxide (MoO_3_) and sulfur powder, bicinchoninic acid (BCA), tetra-n-butylammonium bromide (TBAB), Nafion solution, and ABTS (2,2′-azino-bis(3-ethylbenzthiazoline-6-sulphonic acid)) reagents were purchased from Sigma-Aldrich. (Sigma Aldrich, Saint Louis, MO, USA) Citric acid monohydrate was purchased from Fisher Scientific (Thermo Fischer Scientific, Waltham, MA, USA). Toray Paper (TGP-H-060) was purchased from Fuel Cell Earth (Fuel Cell Earth, Woburn, MA, USA). All reagents were of analytical grade and were used without further purification, and all solutions were prepared using deionized water (18 mΩ·cm).

### 2.2. Laccase Enzymes

A Laccase isoform (EC 1.10.3.2) from *P. sanguineus* CS43 (LacI and LacII) was obtained as described in our previous work [24]. Briefly, mycelia were recovered from a tomato medium supernatant after 10 days of culture by filtration (0.2 μm pore size). Then, the sample was concentrated by ultrafiltration with a tangential-flow filter (Membrane cut-off of 10 kDa, Sartorius Sartojet). The ultra-filtered sample was purified with a DEAE-cellulose ion exchange column eluted with a 20 to 300 mM phosphate buffer of pH 6.0 at a flow rate of 2 mL/min. Finally, laccase fractions were collected and concentrated. Commercially purified laccase from *Trametes versicolor* (EC 1.10.3.2) was kindly provided by Amano Enzyme Inc. (TvL). Laccase activity was determined as in a previous work [37], where the oxidation of 0.54 mM ABTS 50 mM citric/phosphate buffer of pH 4 was monitored by the increase in the absorbance at 420 nm. Total protein content was determined with the BCA reagents using bovine serum albumin (BSA) as standard and measured at 562 nm. Absorbance was measured using a Thermo 50 Scientific^©^ Evolution 260 Bio UV-Visible Spectrophotometer (Thermo Fischer Scientific, Waltham, MA, USA). Measurements were triplicated. All solutions were prepared in MQ water. *P. sanguineus* CS43 fungal laccases were used as obtained from a freshly purified culture, and TvL was prepared according to previous work [37], where it was demonstrated to have an efficient electrochemical signal. Concentrations and/or enzymatic activities were not modified for the following experiments. Characterization of laccase enzymes is shown in Table 1.

### 2.3. Synthesis of MoS_2_ Nanostructured Material

The hydrothermal synthesis of MoS_2_ was performed following the procedure reported by Najmaei et al. [38] with modifications. The modified synthesis is registered under patent MX-a-2017-016742 [39]. The first step consisted of the obtention of MoO_3_ nanoribbons through the hydrothermal method: a dispersion of 2 mL of a saturated ammonium molybdate in HCl mixed by magnetic stirring for 30 min was then transferred to a Teflon-lined autoclave and left at 185 °C for 12 h. MoO_3_ and sulfur powder were put into a tube furnace at 600 °C under nitrogen flow; afterward, the temperature was increased to 800 °C and was heated for 1 h. After the heating process, the furnace was left to cool down to room temperature under nitrogen flow. Then powder was stored for further use.

### 2.4. Immobilization of Laccase onto MoS_2_ Modified Electrodes

Different solutions containing 0.5, 1, 2, and 5 mg/mL of MoS_2_ were prepared and sonicated for 15 min. After a homogeneous solution was obtained, 10 µL of each solution was drop-casted onto the carbon paper (CP) electrode surface (0.25 cm^2^) and left to dry at room temperature (CP-MoS_2_). TBAB-Nafion polymer was used for enzyme immobilization according to the procedure previously reported [40,41]. Briefly, a solution of each purified enzyme (LacI, LacII, and TvL) was mixed with a solution of TBAB-Nafion to obtain a ratio of 3:1 (enzyme: TBAB-Nafion). Then, the solution was vortexed at 2000 rpm for 30 s. After a homogeneous solution was obtained, 10 µL was taken and drop-casted onto (a) pristine electrodes and (b) electrodes modified with MoS_2_ to obtain the CP-Lac and CP-MoS_2_-Lac working electrodes, respectively.

### 2.5. Electrochemical Measurements in the Optimization of Acetaminophen Detection

Voltametric and amperometric measurements were carried out on a CHI 611E Electrochemical Workstation (CH Instruments, Shanghai, China) with a conventional three-electrode system. Either carbon paper (CP) electrode or CP modified electrode was used as the working electrode, saturated calomel as the reference electrode (SCE), and a platinum mesh as the auxiliary electrode. All experiments were conducted at room temperature (~20 °C) in a beaker containing 5 mL of a 0.1 M citric acid/2 M KOH buffer (pH 4). The steady state amperometric response of working electrodes at different ACE concentrations was determined by successive additions of a 66.15 mM of ACE solution in MQ water. First, the working electrodes were equilibrated in 0.1 M citrate buffer at a constant potential of −0.1 V until a constant current was obtained, known as background current (I1). Then, aliquots of the ACE solution were added to the electrochemical cell and the obtained steady-state current response was recorded (I2). The obtained current difference (∆I = I2 − I1) was used to plot a calibration curve of ∆I vs. concentration of dopamine ([ACE]). All measurements were taken by triplicate.

### 2.6. Characterization Techniques

Raman spectra of modified electrodes were acquired using a Renishaw InVia spectrometer under ambient conditions with a 50× objective lens. Laser excitation was 633 nm using an Argon ion laser in the range of 100–1900 cm^–1^. Laser power on the sample was set around 1.0 mW to avoid laser-induced heating. The modification of carbon paper electrodes with MoS_2_ was verified with SEM micrographs. A Nova NanoSEM 200 (FEI Company, Hillsboro, OR, USA) Scanning Electron Microscope with an acceleration voltage of 15 kV in high vacuum conditions with a BSE detector with the capacity to acquire high magnification images (>5000×) was used. Electrochemical impedance spectroscopic (EIS) measurements were carried out on a BioLogic SP-150 potentiostat with a conventional three-electrode system; the frequency range was between 100 kHz and 10 MHz, with the single sine amplitudes of 100 µA.

### 2.7. Application of Optimized Electrodes for the Detection of Acetaminophen in Groundwater Samples

To evaluate the practical effectiveness of the modified electrodes in a real environment, optimal working electrodes were used for the determination of ACE in groundwater. Groundwater samples were obtained from several aquifers located in the state of Nuevo Leon in northeastern Mexico. Sample pH varied between 6.77 and 7.88, indicating neutral to slightly alkaline water conditions within the studied area. Their concentration of major ions, Na^+^, K^+^, Ca^2+^, Mg^2+^, Cl^−^, SO_4_^2−^, NO_3_^−^, and HCO_3_^−^, is related to water–rock interaction (lutites and limestones) [42]. The groundwater samples were pooled, and the pooled sample was preconditioned by adding 50mM citric acid and adjusting pH to 4 with KOH (2M). Electrochemical measurements were performed by successive injections of ACE into a 5 mL preconditioned groundwater sample.

## 3. Results and Discussion

### 3.1. Immobilization of Laccases onto MoS_2_ Modified Electrodes

Michaelis–Menten kinetic parameters of the free and immobilized laccase enzymes on carbon paper electrodes modified with MoS_2_ (CP-MoS_2_-Lac) were determined using ABTS as a substrate. The values of the kinetic parameters shown in Table 2 were obtained by non-linear curve fitting of the reaction rate versus substrate concentration from the Michaelis–Menten equation. As observed, when laccases were subjected to the immobilization process, an increase in *K_Mapp_* of 1.93 and 2.41 was obtained for TvL and LacI. Conversely, this effect was not observed when LacII (35.57 ± 4.79 µM) was immobilized, as no significant changes in its *K_Mapp_* value were observed. The rise in *K_Mapp_* is in general complemented by a decrease in affinity and may be a result of the enzyme’s substrate diffusional restriction or the conformational changes caused by its entrapment in polymer micelles, which prevent substrate and laccase from interacting properly [43,44,45]. These findings indicate the existence and functioning of laccase enzymes after they were immobilized.

**Figure 1:**
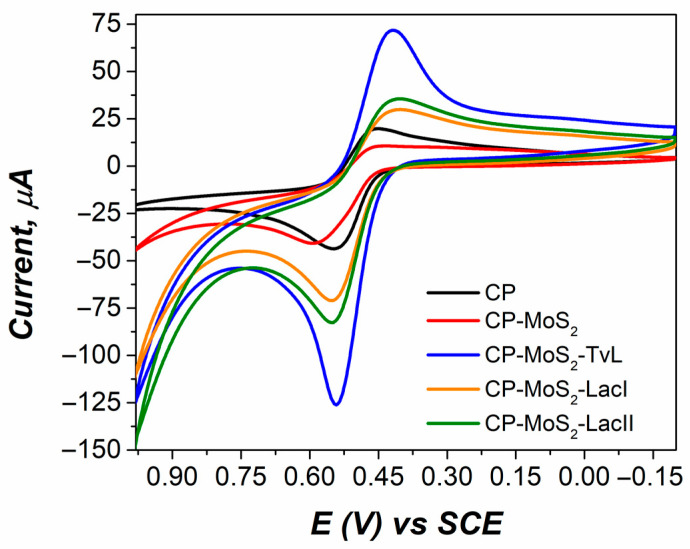
Cyclic voltammograms of electrodes in each immobilization step in a 0.5 mM ACE solution in 0.1 M citrate buffer (pH 4) at a scan rate of 50 mV/s.

Bioelectrocatalytic activity was studied by cyclic voltammetry to corroborate the presence of immobilized laccase enzymes on the electrodes. Figure 1 shows the CVs taken at every film used in the construction of modified electrodes for ACE detection based on the immobilization of laccases onto CP-MoS_2_ electrodes. CVs showed a quasi-reversible system with cathodic and anodic peaks at around 0.45 V and 0.55 V, respectively. Pristine CP presented a reduction peak of 0.45 V and a maximum current of 20 µA; when CP was modified with MoS_2_ (1 mg/mL), current decrease and shift in the reduction peak were observed.

Nonetheless, after adding laccase enzymes to CP-MoS_2_ modified electrodes, it was possible to observe an increase in the current of the cathodic peak for three enzymes of around 30, 36, and 72 µA for LacI, LacII, and TvL respectively. Peak shifting and current increase in peak reduction for all bioelectrodes proved that laccases had been adsorbed on the surface of modified electrode, thus decreasing laccase biocatalytic activity while also causing a slower electron transfer due to the diffusion restriction attained by these layers. All bioelectrodes failed to exhibit a redox reaction in the absence of ACE at potentials between 1.0 and −0.2 V (Supplementary Materials Figure S1).

**Figure 2:**
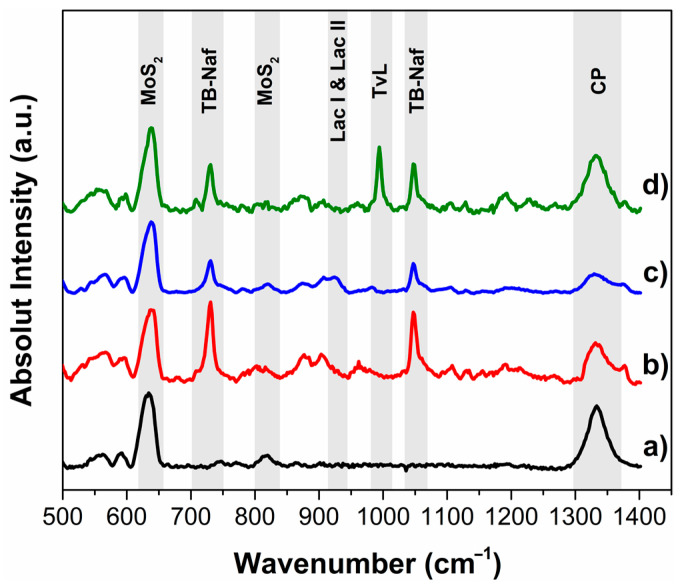
Raman spectra of (a) CP-MoS_2_, (b) CP-MoS_2_-LacI, (c) CP-MoS_2_-LacII, and (d) CP-MoS_2_-TvL.

Laccase immobilization was also studied with Raman spectroscopy; this technique takes advantage of the fingerprint of specific molecules in a sample. Commonly, the analysis of enzymes (proteins) by Raman is based on bands associated with their peptide chains [46]. Figure 2 shows the Raman spectra of laccase-MoS_2_ modified carbon paper electrodes in the range 500–1450 cm^−1^; it was possible to observe characteristic peaks for CP, MoS_2_, TBAB-Nafion, and laccases. Peaks at 1333 cm^−1^ correspond to disorder and defects in the carbon lattice (D-line) from the carbon paper [47]. For MoS_2_, the signal at 645 cm^−1^ corresponds to a combination of the LA(M) frequency and the A1g mode, and the peak at around 820 cm^−1^ is due to the presence of a small amount of MoO_3_ [48,49]. TBAB-Nafion (TB-Naf) presented several peaks in this region. The most prominent peaks at 732 and 1048 cm^−1^ correspond to CF_2_ stretching and sulfonate (SO_3_) symmetric stretch, respectively [50]. Most of the signals for laccase enzymes were overlapped by TBAB-Nafion polymer signals; this condition was expected since it is known that this polymer entraps enzymes through the formation of a semi-permeable membrane that allows the diffusion of substrates and products [51,52]. Nonetheless, LacI and LacII presented a small peak at around 930 cm^−1^ probably for stretching of the γOH in the carboxylic groups in amino acids (glutamic acid and aspartic acid) [53]. TvL showed a more prominent peak at around 995 cm^−1^ probably due to the glycoproteic portion of laccase (C-O ribose); this higher signal may be associated with the fact that TvL was more concentrated than LacI and LacII [54,55].

**Figure 3:**
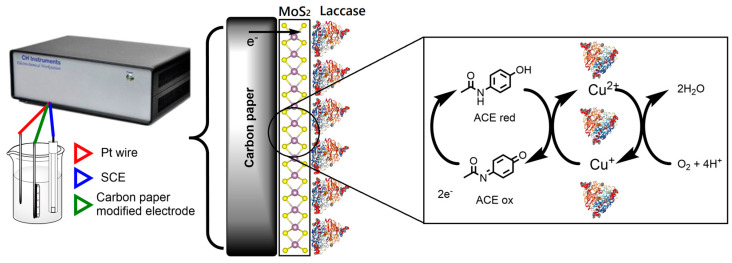
Electron transfer mechanism for our developed CP-MoS_2_ Lac bioelectrodes in the detection of acetaminophen.

The ACE molecule has a hydroxyl and amino derivative functional group; therefore, based on laccase reactivity, the presence of functional groups acting as electron-donating groups (EDG) such as hydroxyl (-OH) and amines (-NH_2_) makes ACE susceptible to laccase attack [56]. The role of MoS_2_ in this mechanism is the modification of the working electrode (carbon paper) to improve its conductive properties (transducer modifier), due to its features as a bandgap ranging from 1.2 to 1.8 eV, as well as higher adsorption capacity of ACEox [57,58,59]. Hence, according to this and CVs results, the proposed electron transfer mechanism for the developed CP-MoS_2_-Lac biosensor is defined as follows: first, acetaminophen (ACEred) is oxidized enzymatically by laccases in the presence of oxygen to its respective N-acetyl-p-benzoquinoneimine form (ACEox), and then ACEox is electrochemically reduced back at the electrode surface to its ACEred form, which is reflected as an electrical current (Figure 3). For amperometric experiments, measurements were made at a potential of −0.1 V to ensure the electrochemical reduction of ACEox. Moreover, this potential avoids possible interference from MoS_2_ precursors, specifically the remaining MoO_3_ (See Figure S3 in Supplementary Materials). This low potential that was used was in accordance with previously reported studies [13,60].

### 3.2. Optimum MoS_2_ Concentration for Laccase Bioelectrode Modification

**Figure 4:**
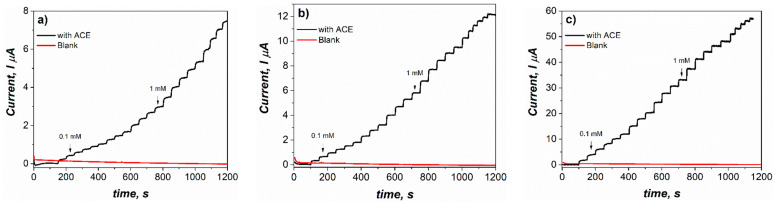
Amperometric i–t curves for the CP-MoS_2_-Lac modified electrodes with successive addition of ACE into 0.1 M citric acid/2M KOH pH 4 recorded at −0.1 V. For (**a**) CP-MoS_2_-TvL, (**b**) CP-MoS_2_-LacI, and (**c**) CP-MoS_2_-LacII.

All nonlinear regression of the Michaelis–Menten model was used to evaluate the apparent electrochemical enzymatic kinetics of the bioelectrodes toward the substrate. This model can be used to study the influence of factors such as temperature, pH, immobilization technique, and diffusion-limiting membranes on an enzymatic system [61,62]. In this sense, the following modified equation was used:

$$J=\frac{J_{max}[S]}{{KM}_{app}+[S]}$$

where *J* is the current density, *K_Mapp_* is the apparent Michaelis constant which quantifies the enzymatic affinity for the substrate, and *J_max_* is the maximum current density [63,64]. Since the recognition system of the proposed enzymatic bioelectrode depends on the enzymatic kinetics, ACE detection was evaluated under steady-state conditions to compare the apparent affinity of *K_Mapp_* and *J_max_* of ACE at the bioelectrodes. Consecutive injections of ACE into a stirred buffer solution measured at a potential of −0.1V were made. Representative amperometric curves for 1 mg/mL of MoS_2_ modified electrodes (Figure 4) showed an evident electrocatalytic effect obtained after adding ACE into the system.

**Figure 5:**
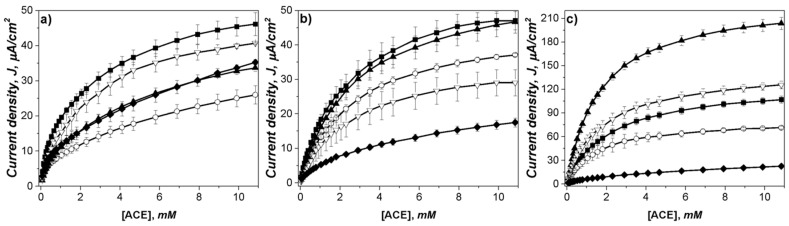
Apparent steady-state Michaelis–Menten kinetics of ACE for (**a**) CP-MoS_2_-TvL, (**b**) CP-MoS_2_-LacI, and (**c**) CP-MoS_2_-LacII, determined in stirred citric acid/KOH (pH 4, 500 rpm) at an applied potential of −0.1 V vs. SCE. MoS_2_ concentrations were: 0 mg/mL (■), 0.5 mg/mL (○), 1 mg/mL (▲), 2 mg/mL (▽), and 5 mg/mL (♦). Error bars represent standard deviation (n = 3).

All the bioelectrodes demonstrated a Michaelis–Menten kinetic behavior, as can be seen in Figure 5. It is also possible to observe that modification of MoS_2_ in a concentration of 0.5–5 mg/mL did not cause a noteworthy increase in the electrochemical detection of ACE when using LacI and TvL, where maximum current densities were around 45 µA/cm^2^. On the other hand, for LacII, a significant increase in current density was observed using 1 and 2 mg/mL of MoS_2_. The maximum current density for LacII was around 210 µA/cm^2^ when using 1 mg/mL of MoS_2_.

Values for *K_Mapp_* and *J_max_* from nonlinear regression of the Michaelis–Menten model are reported in Table 3. Usually, low KM values are associated with high affinity [65]; taking this into account, the results for LacI and TvL modified bioelectrodes confirm that MoS_2_ did not have a significant effect on the concentrations that were studied. Contrarily, for LacII, lower *K_Mapp_* and higher *J_max_* were achieved when 1 and 2 mg/mL of MoS_2_ were used. From these results, the MoS_2_ concentration considered to be optimum for further uses was 1 mg/mL since low *K_Mapp_* and the highest *J_max_* were obtained.

### 3.3. Characterization of Optimum MoS_2_ Modified Electrodes

**Figure 6:**
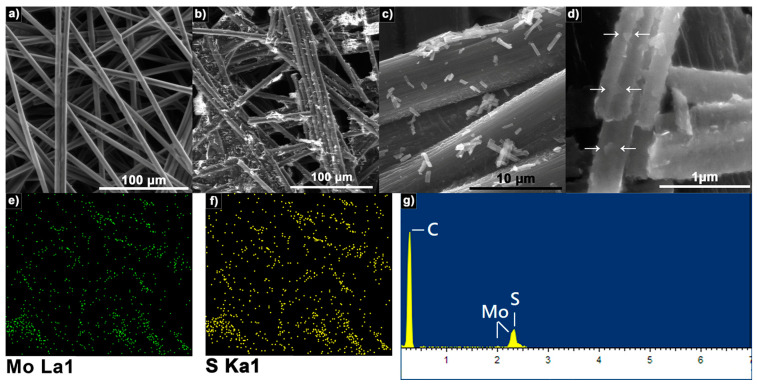
Representative SEM micrographs of electrodes. (**a**) bare CP; (**b**–**d**) CP modified with 1 mg/mL of MoS_2_; (**e**,**f**) EDS mapping of Mo and S elements distribution within CP; (**g**) EDS spectrum of MoS_2_ onto CP.

According to the above results, the electrode with the best efficiency in ACE determination was the one modified with MoS_2_ at 1 mg/mL. SEM and electrochemical impedance spectroscopy (EIS) were carried out to study the electrode properties. SEM micrographs of CP and CP-MoS_2_ modified with 1 mg/mL are shown in Figure 6. From Figure 6a, it is possible to observe that CP is composed of carbon fibers with an average diameter of 6.72 ± 0.65 µm, and that no impurities were observed. When CP was modified with 1 mg/mL of MoS_2_ (Figure 6b), it was well distributed throughout the CP surface; uniform distribution was confirmed by EDS mapping (Figure 6e,f). At high magnification (Figure 6c), it is possible to observe that MoS_2_ morphology resembles ribbons with an average length of 5 µm and an average width of 0.4 µm. Moreover, some nano-platelets grown over the surface (indicated with arrows in Figure 6d) were observed, which can serve as electroactive sites that are more accessible for the substrate to experience reduction reactions. No visible gaps between the stacked MoS_2_ ribbons were visible, suggesting that, as the concentration of ribbon increases, they tend to stack in an ordered manner, ensuring a good contact between adjacent ribbons and the surface of the electrode, which suggests that the electron transfer process is favored. EDS analysis (Figure 6g) was performed to characterize the chemical composition of CP-MoS_2_, confirming the existence of the element C, and Mo and S in a 1:2 ratio.

**Figure 7:**
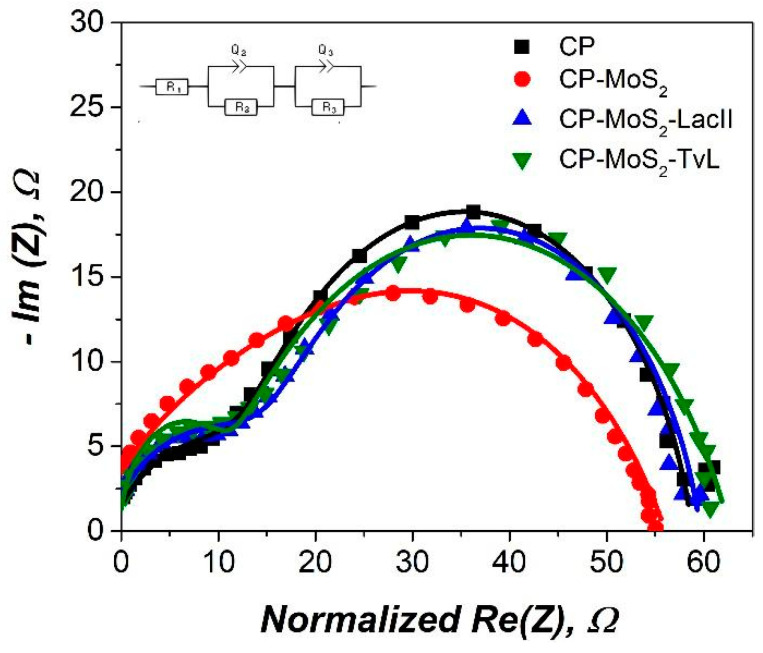
Representative Nyquist plots in each step of bioelectrode fabrication. Measured in citric acid/KOH buffer pH 4 using a frequency range between 100 kHz and 10 MHz, with single sine amplitudes of 100 µA.

The Nyquist plot (Figure 7) shows the electrochemical impedance spectra (EIS) of CP, CP-MoS_2_, CP-MoS_2_-TvL, and CP-MoS_2_-LacII. TvL was taken for comparison. All plots show the typical semicircle portion at high frequencies that correspond to an electron-transfer limited process with the transfer of electrons between the electrolyte and the electrode surface. Symbols represent the experimental data, and solid lines represent the fitted data to an equivalent circuit (inset Figure 7) to determine the charge transfer resistance (Rct) and double-layer capacitance (Cdl) at the interface of the developed electrodes. The basic Randles circuit was modified by an additional parallel RC circuit for a better fit. This additional circuit was required possibly due to the higher roughness of CP [66]. It was possible to observe that CP unmodified by MoS_2_ or laccase had an Rct of 61.21 ± 0.21 Ω. After that, when MoS_2_ was added to the electrode, a slight decrease in Rct to 59.73 ± 0.73 was obtained; this result may be associated with the electrical conductivity given by MoS_2_, promoting direct electron transfer reactions. When TvL was added, an Rct of 63.84 ± 0.62 was obtained, while, for LacII, an Rct to 61.34 ± 0.27 was observed. The hindered electron transfer shown for CP-MoS_2_-TvL and CP-MoS_2_-LacII may be due to the addition of laccase enzymes to the electrode [67]. Despite this increase in transfer-resistant charge compared to CP-MoS_2_, LacII had a lower Rct compared to TvL.

The characterization of electrodes in optimal conditions showed that the use of MoS_2_ as a CP modifier agent in the manufacture of bioelectrodes produces a positive effect, facilitating the transfer of electrons in the system.

### 3.4. Application of Modified Electrodes in the Detection of Acetaminophen

**Figure 8:**
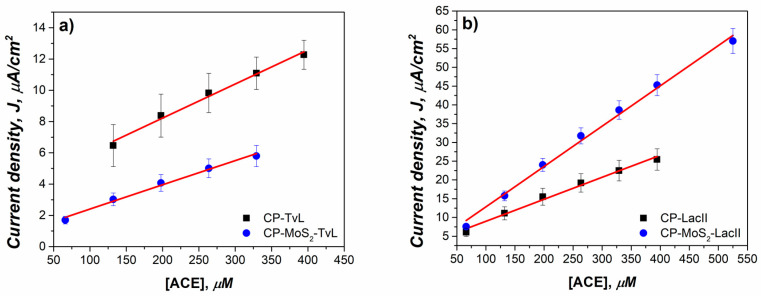
Calibration curves for ACE using the electrodes unmodified and modified with 1 mg/mL of MoS_2_ in citric acid/KOH buffer pH 4 for (**a**) LacII, and (**b**) TvL.

Based on enzymatic kinetics, by using the lower *K_Mapp_* and higher *J_max_* values, the optimal combination of enzyme/MoS_2_ concentration for electrode modification was the one fabricated with LacII and a concentration of 1 mg/mL. Thus, further analyses were performed to determine the linear ranges and limits of detection for ACE. As stated before, commercially available TvL was used for comparison against native LacII. Figure 8 and Table 4 show the results for electrodes unmodified and modified with 1 mg/mL of MoS_2_ and with LacII and TvL.

It was found that CP-MoS_2_-LacII presented a broader linear range of 66–525 µM than TvL, which had a range of 66–329 µM, when both were used in electrodes modified with MoS_2_. These results agree with the fact that the linear range of enzymatic biosensors is limited to substrate concentrations well below Km, where the reaction rate is diffusion-limited and grows almost linearly when substrate concentration increases [68]. The almost 2-fold improvement in sensitivity for the LacII electrode, which passed from 0.0586 to 0.1083 µA/µM cm^2^, can be attributed to the presence of MoS_2_ in the electrode. Consequently, a negative effect of sensitivity could be observed for TvL, which was reduced significantly from 0.0218 to 0.0155 µA/µM cm^2^. The sensitivity values obtained may be associated with changes in enzymatic affinity after immobilization since, as shown above, TvL presented a lower affinity with a decreased sensitivity, whereas LacII affinity was not affected by immobilization process, and exhibited an increase in sensitivity.

The limits of detection (LOD) for CP-MoS_2_-LacII and CP-MoS_2_-TvL were determined by adding a 5 mM acetaminophen solution gradually until a statistically significant change response in current was observed. The obtained values for the modified electrodes were 0.2 µM for CP-MoS_2_-LacII and of 2.00 µM for CP-MoS_2_-TvL, which are among the lowest reported when compared with other oxidoreductase-based electrochemical biosensors for the detection of acetaminophen, as compared in Table 4. In addition, it is possible to observe that our developed bioelectrodes showed better sensitivity in the detection of ACE, reaching almost twice the value of those reported in Table 5. It is important to mention that the amperometric signals recorded for TvL were noisier than those recorded by the native LacII enzyme; this fact could translate into a much more reliable measurement.

### 3.5. Application of Modified Electrodes in the Amperometric Detection of Acetaminophen in a Groundwater Sample from a City in Northeastern Mexico

With the aim of confirming the validity and feasibility of the biosensor for detecting ACE in groundwater, an aliquot of groundwater was preconditioned by adding citric acid to reach a concentration of 50 mM, then adjusting pH to 4 with KOH. Then, successive injections of ACE were made to a 5 mL preconditioned groundwater sample. The detection of ACE was studied by increasing concentration up to around 150 µM to determine the linear range, sensitivity, and LOD in this water sample used as a model of environmental importance.

**Figure 9:**
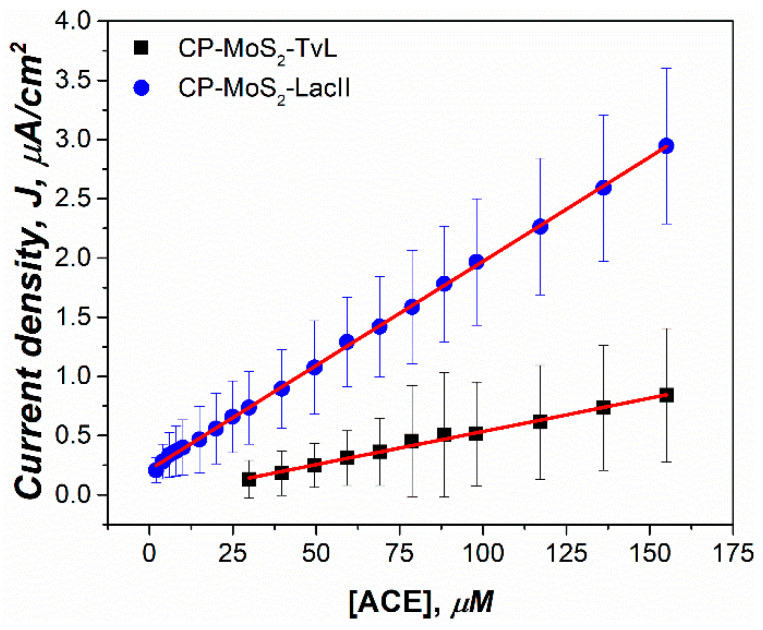
Calibration curves for ACE using the electrodes modified with 1 mg/mL of MoS2 for LacII and TvL, both in groundwater (5 mL) pre-conditioned with 0.05 M citric acid and adjusted to pH 4 con 2M KOH.

Figure 9 and Table 6 summarize the application of 1 mg/mL modified electrodes with TvL and LacII in the detection of ACE in groundwater; this study was performed up to a concentration of around 150 µM, where typical CECs are found [8,74].

LacII presented a wider linear range from 1 to 155 µM compared to TvL, which had a linear range of 29.82–155 µM. In terms of linearity, LacII also presented a better R^2^ value of 0.9992 than the TvL (0.9933). As is also shown in Table 6, LOD for Lac II was lower than that for TvL, which was an expected result since, on the one hand, it is known that Lac II has presented an enhanced resistance to common inhibitors such as major ions Na^+^, K^+^, Ca^2+^, Mg^2+^, Cl^−^, SO_4_^2−^, NO^3−^, which are normally found in groundwater, and, on the other hand, LacII did not show significant affinity change after the immobilization process. Furthermore, the sensitivity of LacII was shown to be around 3-fold higher when compared to TvL, confirming that the native laccase enzyme is a better candidate for use in practical applications for ACE detection in water samples of environmental importance such as groundwater.

From these results, it was possible to observe that the use of native laccase LacII for the fabrication of a modified electrode in the development of a laccase-based electrochemical biosensor can be a powerful element for the determination of any noticeable increase in the guidance value for ACE of 200 µg/L, which can be an indicator of pollution by this drug. Moreover, laccase-based amperometric biosensors have the advantage of being of easy usage, fast, and with good performance and selectivity, which may be convenient for the detection of these emerging pollutants [75].

## 4. Conclusions

The use of MoS_2_ as a two-dimensional material for the modification of carbon paper electrodes coupled with the immobilization of laccase enzymes allowed the development of a novel bioelectrode for use in the development of a laccase-based amperometric biosensor. The resulting bioelectrode showed good analytical performance in the determination of ACE in citric acid buffer, providing a sensitivity of 108.3 nA/µM∗cm^2^ and an LOD of 0.2 µM, which are among the lower values reported for this oxidoreductase-based biosensor. It was also demonstrated that MoS_2_ allowed an increase in sensitivity. Furthermore, the difference in the nature of laccase enzymes was investigated, presenting a very important effect for the development of novel bioelectrodes; hence, it is of utmost importance to investigate enzymes with improved detection yields. For example, of the two isoforms of the laccase enzyme from the white-rot fungi, *Pycnoporus sanguineus* CS43 (LacI and LacII) and the commercial laccase (*Trametes versicolor*), better results were achieved for LacII native laccase.

The bioelectrode application in the determination of ACE in groundwater samples was successfully carried out, with LacII showing a good performance against naturally found inhibitors (major ions). This achieved an LOD of 0.5 µM and a linear range of 1–98.04 µM equivalent to 151.13–14,819.9 µg/L. The properties of this developed bioelectrode can be effectively used to develop a monitor tool for any significant increase in guidance values emitted by environmental organisms defined at 200 µg/L to study water pollution by this pharmaceutical. In a field in which few studies have been carried out, this work contributes new findings in the application of these types of electrochemical sensors in environmental samples [60].

## Figures and Tables

**Table 1:** Enzymatic characterization of laccases from *P. sanguineus* CS43 and *Trametes versicolor*.

Sample	Conc. (mg/mL)	Activity (U/mL)	Specific Activity (U/mg)
TvL	1.32 ± 0.64	81.00 ± 30	61.50 ± 3.29
LacI	0.31 ± 0.48	33.87 ± 1.38	108.17 ± 7.35
LacII	0.48 ± 0.17	74.49 ± 0.97	155.11 ± 1.50

**Table 2:** Michaelis-Menten kinetic constants for both free and immobilized laccase enzymes on the carbon paper electrode surface.

Laccase	*K_Mapp_*	
	Free	Immobilized
TvL	42.07 ± 3.136	123 ± 26.96
LacI	16.64 ± 2.548	58.68 ± 10.12
LacII	34.51 ± 5.857	35.57 ± 4.79

**Table 3:** Apparent steady-state Michaelis–Menten kinetic values of LacI, LacII, and TvL MoS_2_ modified bioelectrodes for ACE, determined in stirred citric acid/KOH (pH 4, 50 mM, 500 rpm) at an applied potential of −0.1 V vs. SCE.

MoS_2_	TvL		LacI		LacII	
Concentration	*K_Mapp_* (µM)	*J_max_* (µA/cm^2^)	*K_Mapp_* (µM)	*J_max_* (µA/cm^2^)	*K_Mapp_* (µM)	*J_max_* (µA/cm^2^)
**Unmodified**						
-	1659 ± 250	51.11 ± 2.59	2164 ± 151	53.68 ± 1.43	1908 ± 77	129.3 ± 1.76
**Modified with MoS_2_**						
0.5mg/mL	2613 ± 254	29.80 ± 1.09	2474 ± 65	45.39 ± 0.44	1501 ± 99	81.05 ± 1.68
1 mg/mL	2952 ± 155	41.03 ± 0.85	2702 ± 143	58.01 ± 1.17	1656 ± 37	234.7± 1.65
2 mg/mL	1791 ± 99	49.84 ± 0.91	2429 ± 232	34.34 ± 1.30	1604 ± 66	142.2± 1.81
5 mg/mL	2733 ± 253	40.33 ± 1.43	3535 ± 250	22.07 ± 0.65	4176 ± 331	29.54 ± 1.04

**Table 4:** Linear range, sensitivities, and correlation coefficients obtained for the calibration curves of the modified electrodes in the determination of ACE.

Bioelectrode	Linear Range (µM)	Sensitivity (µA/µM cm^2^)	R^2^
CP-LacII	66–395	0.058	0.9901
CP-MoS_2_-LacII	66–525	0.108	0.9948
CP-TvL	132–395	0.021	0.9901
CP-MoS_2_-TvL	66–329	0.015	0.9901

**Table 5:** Analytical characteristics of some enzyme-based electrochemical biosensors reported for the determination of acetaminophen.

Electrode *	Detection Method	Sample	Linear Range (µM)	Sensitivity (µA/µM cm^2^)	LOD (µM)	Ref.
GCPE-PPO	Amp (−0.1 V)	50 M phosphate buffer solution pH 7.4.	Up to70	0.015 µA/µM	7.8	[60]
GCE-HRP @ PAA-BIS	Amp (−0.1 V)	50 mM sodium phosphate buffer pH 7.0 with 100 mM KCl	5.6–331.1	0.069	1.7	[13]
GCE-HRP-PPY SPE-HRP-PPY	Amp (−0.175 V)	0.2 mM H_2_O_2_ in phosphate buffer pH = 7.4	9.3–83.7 3.1–55.9	- -	6.5 1.52	[69]
GCE-nano PPY-HRP GCE-flat PPY-HRP	Amp (−0.2 V)	Phosphate buffer solution (pH 7.4)	5–60 5–300	0.050 0.002	0.1 4.1	[70]
GCE-clay-PEI-HRP	Amp (0 V)	100 mM phosphate buffer saline (pH 7.4)	5.25–49.5	0.013 µA/µM	0.63	[71]
SPE-CoPC/Tyr	CV	100 m M phosphate-buffer pH 6/100 mM KCl	up to 40	−0.088	0.5	[15]
CPE-EP-PPO	DPV 10 mV/s	100 mM phosphate buffer (pH 6.0)	600–1150	-	5.0	[72]
CPE-PPO	DPV 10 mV/s	100 mM phosphate buffer pH 7.0	5–245	-	3.0	[73]
CP-LacII	Amp −0.1 V	100 mM citric acid/2 M KOH buffer (pH 4)	66–395	0.058	-	Present study
CP-TvL			132–395	0.021	-	
CP-MoS_2_-LacII			66–525	0.108	0.2	
CP-MoS_2_-TvL			66–329	0.015	2.0	
CP-MoS_2_-LacII	Amp −0.1 V	Groundwater (50 mM citric acid/2 M KOH)	1–155.1	0.017	0.50	Present study
CP-MoS_2_-TvL			29.82–155.1	0.005	24.88	

* GCPE-: glassy carbon paste electrode. PPO: polyphenol oxidase. GCE: glassy carbon electrode. HRP horseradish peroxidase. PAA-BIS:—polyacrylamide—*N*,*N*-methylenebisacrylamide. PPY: polypyrrole. SPE: screen-printed electrode. PEI: polyethyleneimine. CoPC: polyvinyl alcohol photocrosslinkable polymer. Tyr: tyrosinase. CPE: carbon paste electrode. EP-PPO: eggplant polyphenol oxidase. MWCNT: multiwall carbon nanotubes. PANI: polyaniline. GA: glutaraldehyde. Amp: Amperometry. CAmp: Chronoamperometry. CV: Cyclic Voltammetry. DPV: Differential pulse voltammetry.

**Table 6:** Linear range, sensitivities, and correlation coefficients obtained for the calibration curves for the modified electrodes in the determination of ACE in groundwater samples.

Electrode	Linear Range (µM)	Linear Range (µg/L)	Sensitivity (nA/µMcm^2^)	R^2^	LOD (µM)	LOD (µg/L)
CP-MoS_2_-Lac	1–155	151.13–23,436.1	17.7	0.9992	0.50	75.57
CP-MoS_2_-TvL	29.82–155	4507.84–23,436.1	5.6	0.9933	24.88	3760.27

## Data Availability

Data available on request.

## Supplementary Materials

The following supporting information can be downloaded at: https://www.mdpi.com/article/10.3390/s23104633/s1, Figure S1: CVs of developed bioelectrodes in the absence of ACE; Figure S2: Representative SEM images of the as obtained MoS_2_ nanostructured material; Figure S3: Cyclic voltammograms of modified electrodes using MoS_2_-R and MoO_3_ in the absence of immobilized enzymes and of ACE; Figure S4: Enzyme leaking test. Refs. [41,45,52] are cited in Supplementary Materials.
