# Supplementary material for: Detection of Acetaminophen in Groundwater by Laccase-Based Amperometric Biosensors Using MoS2 Modified Carbon Paper Electrodes
Source: Sensors (Basel). 2023 May 10;23(10):4633. doi: 10.3390/s23104633 (PMC10222279; doi:10.3390/s23104633)
Supplement: Supplementary file 1 [file sensors-23-04633-s001.zip › sensors-2358757-supplementary.pdf]

Supplementary information

Detection of acetaminophen in groundwater by laccase based amperometric biosensors using MoS_2 modified carbon paper electrodes.

Marcela Herrera-Domínguez[1], Koun Lim[2], Iris Aguilar-Hernández[1], Alejandra García-García[3], Shelley D. Minteer[2], Nancy Ornelas-Soto[1]*, Raúl García-Morales[4]**

[1] Laboratorio de Nanotecnología Ambiental, Escuela de Ingeniería y Ciencias, Tecnológico de Monterrey, Ave. Eugenio Garza Sada 2501, Monterrey 64849, NL, Mexico.

[2] Department of Chemistry and Materials Science & Engineering, University of Utah, Salt Lake City, UT 84112, USA.

[3]Laboratorio de Síntesis y Modificación de Nanoestructuras y Materiales Bidimensionales, Centro de Investigación en Materiales Avanzados S.C., Unidad Monterrey, Parque PIIT, Apodaca 66628, NL, Mexico.

[4]Centro de Nanociencias y Nanotecnología, Universidad Nacional Autónoma de México, Carretera Tijuana-Ensenada Km. 107, Ensenada 22860, BC, Mexico.

* Correspondence: ornel@tec.mx (N.O.-S.); raul.garmo@ens.cnyn.unam.mx (R.G.-M.)

Supplementary Information S1

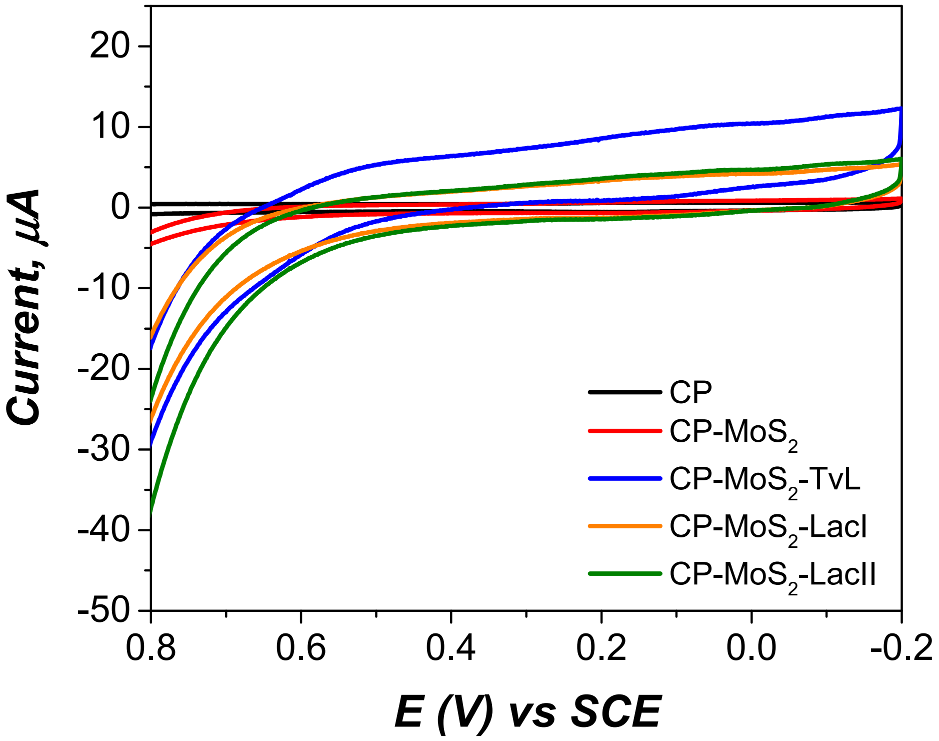

Figure S1. CVs of developed bioelectrodes in the absence of ACE.

Supplementary Information S2

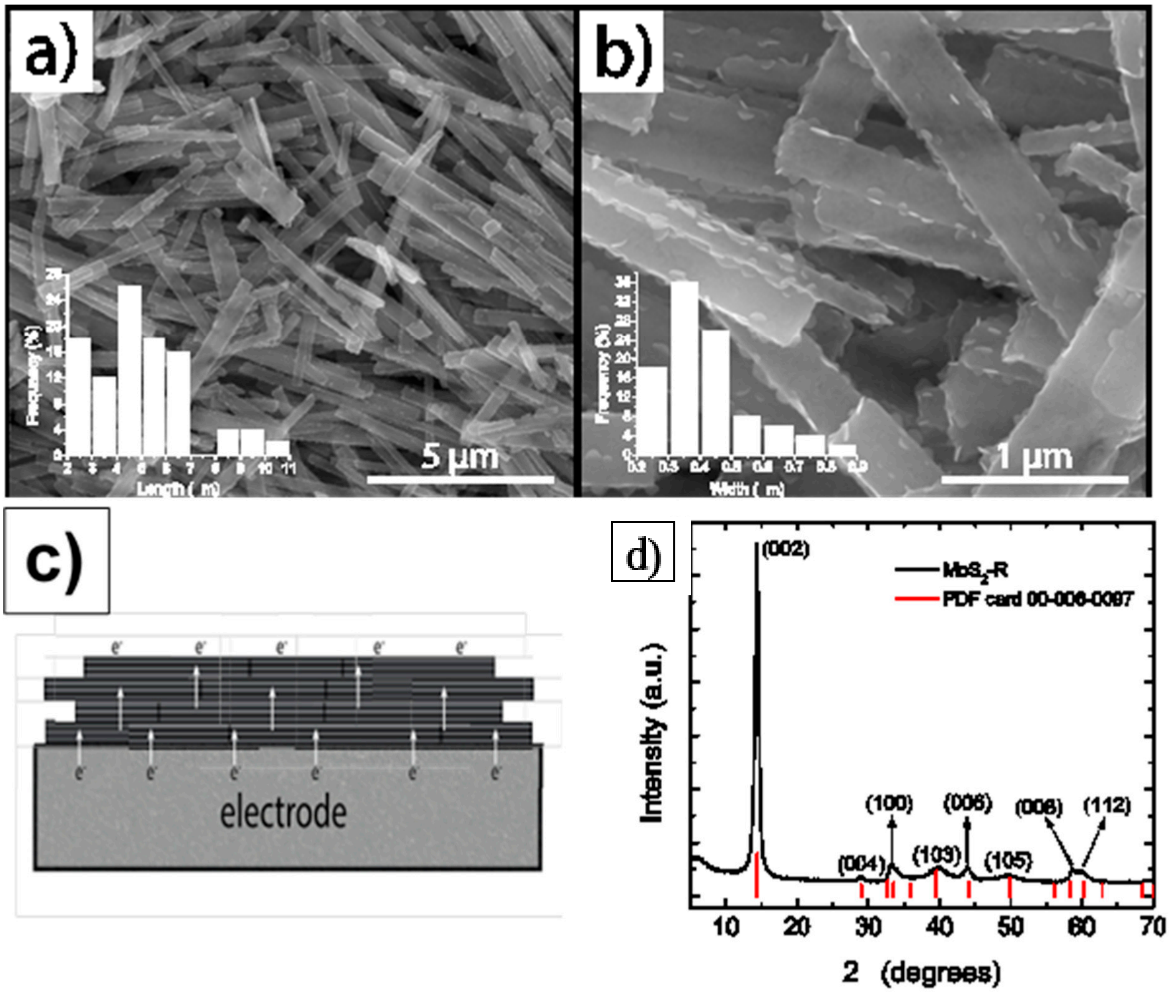

Figure S2. Representative SEM images of the as obtained MoS_2 nanostructured material (a, b). Schematic representation of the electrodes modified with MoS_2-R (c) and X-ray diffraction patterns (XRD) of MoS_2 (d)

Supplementary Information S3

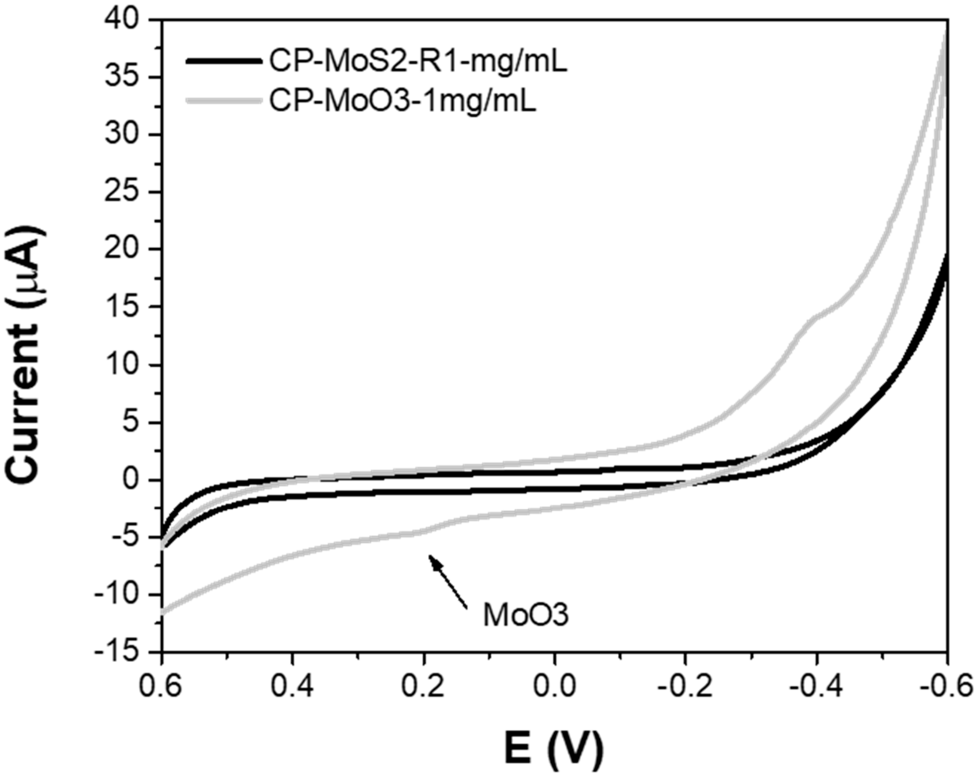

Figure S3. Cyclic voltammograms of modified electrodes using MoS2-R (1 mg/mL) and MoO3-(1 mg/mL) in the absence of immobilized enzymes and of ACE. Measurements were recorded in 5 mL of solution pre-conditioned with 0.05 M citric acid and adjusting to pH 4 con 2M KOH.

Supplementary Information S4

The Figure S4 shows the behavior of each electrode (Lac II and TvL) over 5 use-cycles. In the first cycle, the enzyme activity released was around 5% for all enzymes used, in the second use 0.1 % of activity was detected. These results can be due to the fact that some enzymes remained free during the immobilization process using the TBAB-Nafion polymer, this behavior is similar to previously published works [1]. For further cycles, no activity was detected, demonstrating that laccases were stably retained in the electrode by the TBAB-Nafion polymer [2,3].

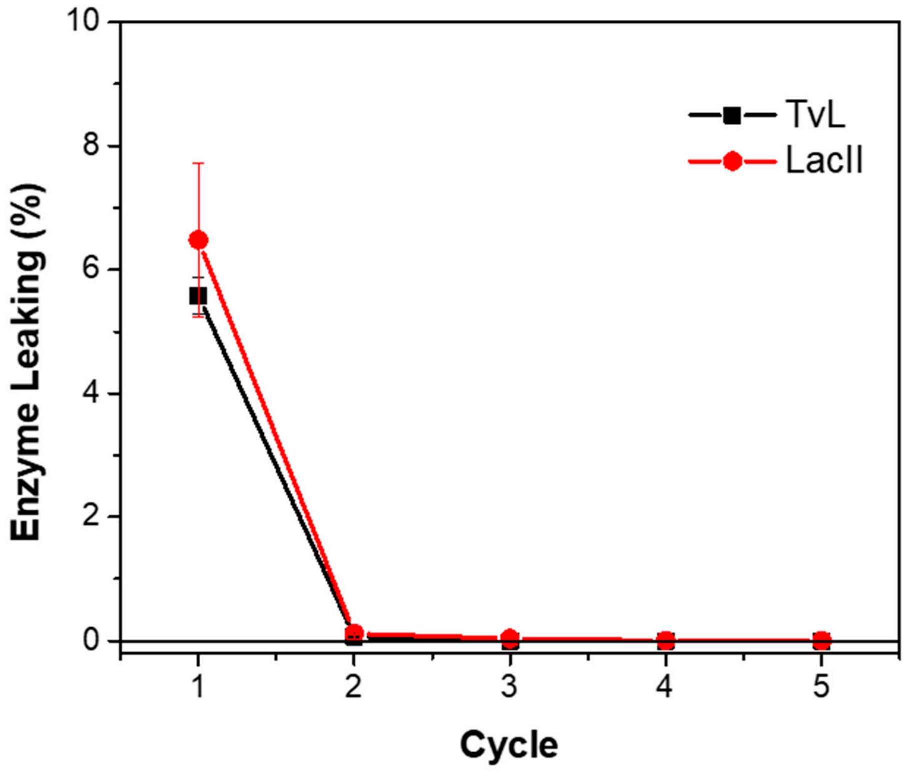

Figure S4. Enzyme leaking test.

References

1. Batra, B.; Yadav, M.; Pundir, C.S. L-Glutamate Biosensor Based on l-Glutamate Oxidase Immobilized onto ZnO Nanorods/Polypyrrole Modified Pencil Graphite Electrode. *Biochem. Eng. J.* **2016**, *105*, 428–436, doi:10.1016/j.bej.2015.10.012.

2. Mailley, P.; Cummings, E.A.; Mailley, S.; Cosnier, S.; Eggins, B.R.; McAdams, E. Amperometric Detection of Phenolic Compounds by Polypyrrole-Based Composite Carbon Paste Electrodes. In Proceedings of the Bioelectrochemistry; Elsevier, June 1 2004; Vol. 63, pp. 291–296.

3. Meredith, S.; Xu, S.; Meredith, M.T.; Minteer, S.D. Hydrophobic Salt-Modified Nafion for Enzyme Immobilization and Stabilization. *J. Vis. Exp.* **2012**, e3949, doi:10.3791/3949.
